# Research Progress on Major Influencing Factors of Corrosion Behavior of Pipeline Steel in Supercritical CO_2_ Environment

**DOI:** 10.3390/ma18112424

**Published:** 2025-05-22

**Authors:** Zhe Liu, Qian Gao, Yong Zhou, Ruijuan Pan

**Affiliations:** School of Materials Science and Engineering, Xi’an Shiyou University, Xi’an 710065, China

**Keywords:** CCUS, supercritical CO_2_, corrosion, welded joint

## Abstract

Carbon capture, utilization and storage (CCUS) represents a vital technological strategy for mitigating greenhouse gas emissions and facilitating sustainable development. Supercritical CO_2_ (SC-CO_2_) pipeline transportation serves as an essential intermediary step towards attaining the “Dual Carbon Goals” and CCUS, representing the optimal and most cost-effective solution for ultra-long distance transport. In the CO_2_ capture process, trace amounts of impurities, such as H_2_O, O_2_, H_2_S, NO_x_ and SO_x,_ are inevitable. These gases react to form acidic compounds, thereby accelerating pipeline corrosion. With the progression of CCUS initiatives, corrosion within supercritical CO_2_ pipeline transportation has become a critical challenge that significantly affects the safety and integrity of pipeline infrastructure. This review paper provides an in-depth analysis of the corrosion behavior of pipeline materials in a supercritical CO_2_ environment, with particular attention to the effects of impurity, temperature, and pressure on corrosion rates, corrosion products, and corrosion morphology. Furthermore, an analysis of the corrosive behavior of welded joints in supercritical CO_2_ transport pipelines is performed to provide valuable reference data for research and construction projects related to these pipelines.

## 1. Introduction

With the acceleration of industrialization and the ongoing expansion of human activities, greenhouse gas emissions have consistently risen, resulting in substantial modifications to the Earth’s climate system. Global warming has emerged as one of the most pressing challenges confronting our world today. To achieve sustainable development goals and tackle global climate change, 178 parties collectively adopted the Paris Agreement at the 2015 United Nations Climate Change Conference, aiming to collaboratively mitigate the accelerating trend of global warming [1,2]. In 2020, China launched the ambitious “Dual Carbon Goals”, which aim to achieve peak carbon emissions by 2030 and carbon neutrality by 2060 [3,4]. CCUS technology represents an effective approach to mitigate greenhouse gas emissions from fossil fuel usage, integrating the processes of CO_2_ capture, transportation, utilization, and storage [5,6]. According to the 2024 Global Status of CCS report by the Global CCS Institute, 2024 has seen significant growth in CCS facility development. As of July 2024, the pipeline includes 628 projects, a 60% year-on-year increase. Capture capacity has seen strong growth since 2017, with an annual compound rate of 32% [7]. Efficient transportation of CO_2_ is critical for integrating capture and utilization within the CCUS system. Presently, high-pressure pipeline transportation of CO_2_, operating at pressures between 2 and 15 MPa, represents the most cost-effective and efficient method for ultra-long-distance transport [8]. The number of pipeline projects has seen a substantial increase. Currently, the global CO_2_ pipeline network extends over 8000 km in length. Projections suggest that, by 2050 and beyond, more than 200,000 km of pipelines will be necessary to support the annual transportation of approximately 10 Gt of CO_2_ [9]. In August 2022, China inaugurated its first large-scale CCUS project with the establishment of a 109-km CO_2_ transport pipeline linking Qilu Petrochemical and Shengli Oilfield. The pipeline is engineered to withstand a pressure of up to 12 MPa [10,11].

**Figure 1:**
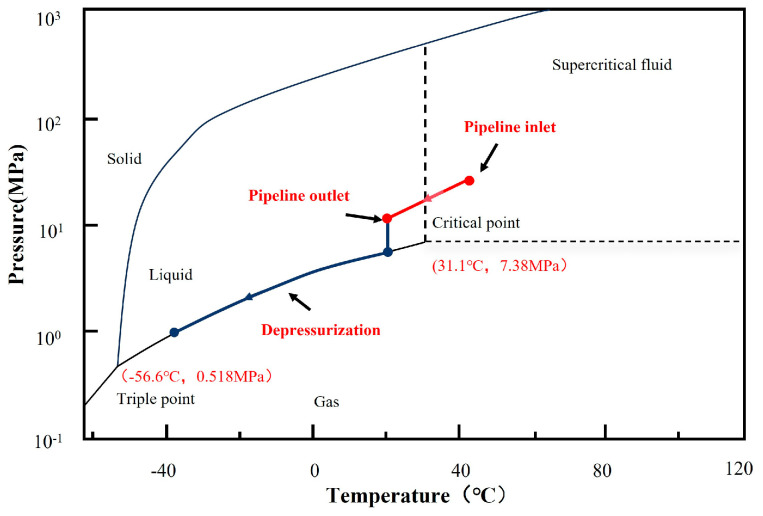
Pure CO_2_ phase diagram [14].

To enhance cost-effectiveness and optimize transportation efficiency, CO_2_ is typically compressed into its supercritical state, referred to as SC-CO_2_. Pure CO_2_ has a triple point at −56.6 °C and 0.518 MPa, which determines the point where CO_2_ may co-exist in gas, liquid and solid state [12]. The CO_2_ critical point is 7.38 MPa and 31.1 °C; below the critical temperature, the CO_2_ is in the liquid dense phase, and above in the supercritical phase. There is no noticeable phase change above the critical temperature, hence, when the pressure is reduced from above to below the critical pressure, a smooth enthalpy change occurs from super critical fluid to gas [12,13]. The phase diagram for pure CO_2_ as illustrated in Figure 1 [14]. In the supercritical state, neither an increase in pressure nor temperature results in a significant alteration of the state of CO_2_. The unique properties of supercritical CO_2_, such as low viscosity and high density, render it an energy-efficient state for transportation [14,15,16,17]. However, operating at higher pressures is associated with increased risks of corrosion and ductile running fractures of pipeline. The underground installation of supercritical CO_2_ transport pipelines demands the utmost emphasis on pipeline safety. Potential leaks could lead to significant adverse outcomes, including greenhouse gas emissions, water contamination, soil acidification, and considerable economic losses [17,18]. The existing review papers address various aspects, including the design of CO_2_ transportation pipelines, evaluation methods for metal corrosion in dense-phase CO_2_ environments, the corrosion mechanisms of pipelines, and anti-corrosion technologies. Considering the internal corrosion of pipeline steel in a supercritical CO_2_ environment, this study comprehensively examines the influence of various factors, including material composition, moisture, impurities, temperature, pressure, and flow rate, on corrosion rate, corrosion products, and corrosion morphology. Furthermore, this study reviews the current research status of welded joints under supercritical CO_2_ conditions, offering valuable insights for investigating corrosion issues in supercritical CO_2_ transport pipelines.

## 2. Corrosion Behavior of Carbon Steel in CO_2_

**Figure 2:**
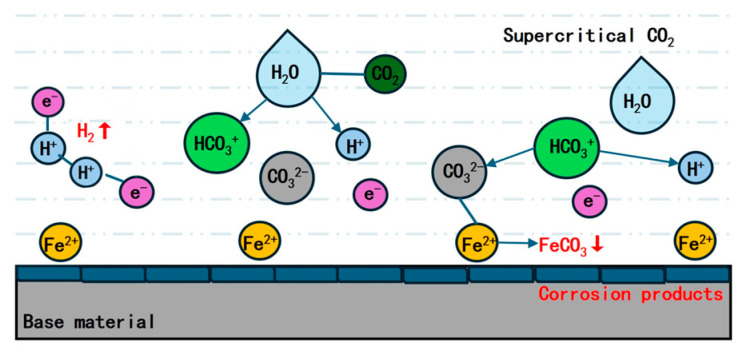
Schematic diagram of corrosion.

CO_2_ corrosion results from the dissolution of CO_2_ in water, which subsequently forms carbonic acid (H_2_CO_3_). The interaction between carbon steel and CO_2_ triggers a complex electrochemical corrosion process, which ultimately results in corrosion on the metal surface. The reaction mechanism responsible for this phenomenon is illustrated as follows [19,20,21,22]. The corrosion schematic is shown in Figure 2.

(1) When CO_2_ dissolves in water, it reacts chemically with water molecules to form H_2_CO_3_. H_2_CO_3_ is categorized as a weak acid, and a fraction of it further dissociates into bicarbonate ions (HCO_3_^−^) and carbonate ions (CO_3_^2−^).
  (1) $${CO}_{2}\left(g\right)↔{CO}_{2}\left(aq\right)$$
  (2) $$CO_{2}\left(g\right)+H_{2}O\left(l\right)↔H_{2}CO_{3}\left(aq\right)$$
  (3) $$H_{2}CO_{3}↔H^{+}+HC{O_{3}}^{-}$$
  (4) $$HC{O_{3}}^{-}↔H^{+}+{CO_{3}}^{2-}$$
(2) Cathode reaction: The reduction of H+ ions occurs, leading to the acquisition of electrons and the formation of H_2_. Additionally, this reduction process involves H_2_CO_3_ and CO_3_^2−^.
  (5) $$2H^{+}+2e^{-}↔H_{2}$$
  (6) $$2H_{2}CO_{3}+2e^{-}\to H_{2}+2HC{O_{3}}^{-}$$
  (7) $$2HC{O_{3}}^{-}+2e^{-}\to H_{2}+2{CO_{3}}^{2-}$$
(3) The anode reaction encompasses the dissolution of the Fe anode, the generation of Fe^2+^ ions, and the formation of carbonate compounds.
  (8) $$Fe\to {Fe}^{2+}+2e^{-}$$
  (9) $${Fe}^{2+}+{CO_{3}}^{2-}\to FeCO_{3}$$
  (10) $${Fe}^{2+}+2HC{O_{3}}^{-}\to {Fe\left(HCO_{3}\right)}_{2}$$
  (11) $${Fe\left(HCO_{3}\right)}_{2}\to FeCO_{3}+CO_{2}+H_{2}O$$

The substrate surface is coated with a dense film of corrosion products (FeCO_3_), which effectively inhibits the diffusion of corrosive ions. This protective layer consequently safeguards the material surface and mitigates the corrosion process initiated by the corrosive medium, ultimately enhancing the overall corrosion resistance.

The corrosion of steel in CO_2_ environment can be classified into localized and uniform corrosion [23]. Currently, there is no unified regulation regarding corrosion standards for CCUS supercritical CO_2_ pipelines in China. However, within the petroleum industry, severe corrosion is defined as a uniform corrosion rate exceeding 0.25 mm/year for carbon steel and a maximum pitting depth greater than 0.38 mm/year [24]. The corrosion environment can be primarily categorized into two phases: the water-saturated CO_2_ phase, which predominantly occurs during CO_2_ transport and injection, and the CO_2_-saturated water phase, which is mainly observed during CO_2_ injection and subsequent oil and gas production [25,26].

## 3. Factors Influencing Corrosion in Supercritical CO_2_ Transportation Pipelines

The corrosion issues in supercritical CO_2_ pipelines primarily originate from two factors. First, in the carbon capture process, despite achieving a CO_2_ purity of over 99.5%, the captured CO_2_ frequently contains trace amounts of impurities, such as H_2_O, O_2_, NO_x_, H_2_S, and SO_x_ [7,27,28]. Internal corrosion is a significant risk to the integrity of carbon steel pipelines in case of insufficient dewatering of the CO_2_ composition [14]. Once a water droplet is formed and attached to the steel surface, it strongly etches the surface and initiates localized attacks, potentially leading to leakage [15]. These impurities, such as NO_x_, H_2_S or SO_x,_ can dissolve in water, leading to a decrease in pH levels and an increase in the corrosion rate of pipeline steel. In severe cases, this may result in material failure and CO_2_ leakage, posing potential safety hazards. The second factor pertains to the impact of internal conditions during transportation, such as multiphase flow (coexistence of gas and liquid phases) induced by fluctuations in temperature and pressure. These conditions can result in localized corrosion [29,30,31]. In summary, the corrosion characteristics of CO_2_ are influenced by multiple factors, including material composition, impurity content, CO_2_ partial pressure, temperature, pH value, and flow rate. These factors can potentially accelerate the corrosion rate of pipelines, thereby shortening their service life. American Petroleum Institute (API) specification 5L is extensively utilized in the classification and designation of pipeline steel grades. Therefore, the materials examined in the literature on the corrosion of pipeline steel in supercritical CO_2_ environments primarily comprise specific grades of pipeline steel, such as X65 and X70.

### 3.1. Water Content

Among various impurities, H_2_O is the most significant factor. H_2_O not only reacts directly with Fe as a gaseous oxidizer but can also condense on the steel surface, dissolve CO_2_ or other impurity gases, and form an acidic solution. This process facilitates electrochemical reactions, thereby exacerbating corrosion damage [32]. For instance, when SO_2_ impurities are present, reducing the water content is more effective in mitigating corrosion issues compared to decreasing the SO_2_ concentration [33]. In scientific studies, water content is typically expressed in two primary forms: absolute water content and relative humidity. The actual water content is typically quantified in parts per million by volume (PPMV), whereas relative humidity (RH) is commonly expressed as a percentage (%). RH represents the ratio of the actual water content to the saturation water content, which is influenced by both temperature and pressure [34]. Table 1 presents a comprehensive summary of numerous authors’ results from their experiments in supercritical CO_2_ environments with differing water content levels.

Jiang et al. [35] investigated the corrosion behavior of X65 steel in various CO_2_ environments and discovered that the critical water content for initiating corrosion is 60% RH in both liquid and supercritical CO_2_, whereas this is 80% RH in gaseous CO_2_. Prior to the observation of a single water phase, the corrosion rate remains stable as relative humidity increases. When the relative humidity exceeds 60% at temperatures of 35 °C and a pressure of 8 MPa, localized corrosion and severe uniform corrosion will occur. Hua et al. [36] investigated the corrosion behavior of X65 steel exposed to supercritical CO_2_ at 50 °C and 80 bar. The average corrosion rate, after immersing the sample in 300 mL of supercritical CO_2_-saturated water for 96 h, was measured to be 4.1 mm/year. In the supercritical CO_2_ phase saturated with water (exceeding 3400 ppm), an FeCO_3_ corrosion product film gradually formed and densified on the sample surface over time, leading to a progressive reduction in the corrosion rate. The corrosion rate was measured at 0.03 mm/year, whereas local corrosion rates were observed to reach up to 1.4 mm/year for immersion times of 48 h. Reducing the water content to 2650 ppm resulted in a decrease in the corrosion rate to 0.015 mm/year and a mitigation of the local corrosion rate to 0.2 mm/year. Notably, no dissolution of the steel was observed when the water content was below 1600 ppm. Liu et al. [37] constructed a novel in situ electrochemical noise (EN) testing system for supercritical CO_2_ corrosion, and the influences of water content (500–5000 ppmv) on the corrosion kinetics of X65 pipeline steel were investigated. The results revealed that the primary corrosion type was localized corrosion, characterized by the coexistence of metastable and stable pitting, with a trend toward uniform corrosion. Both the uniform corrosion rate and pitting corrosion rate were found to increase when increasing the water content, and the pitting corrosion rate was almost 20 times more than the uniform corrosion rate.

**Figure 3:**
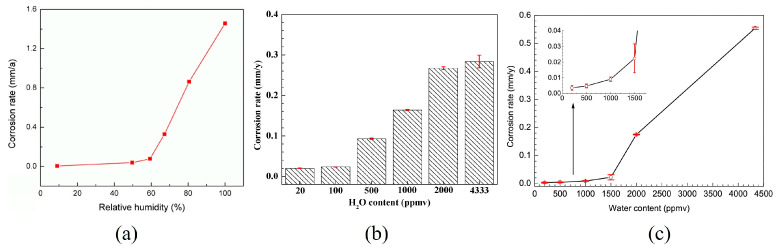
(**a**) The variation in the corrosion rates of X70 steel with relative humidity [38]; (**b**) Variations in corrosion rate of X52 steel with H_2_O content exposed to supercritical CO_2_ steams containing the impurities of O_2_, H_2_S, SO_2_ and NO_2_ at 10 MPa and 50 °C for 72 h [39]; (**c**) Variation in water content on corrosion rate of X65 at 50 °C and 10 MPa [40].

The critical relative humidity varies across different systems. Xiang et al. [38] quantified the corrosion rate of X70 steel in a CO_2_-SO_2_-O_2_ environment using the weight loss method, and developed a thermodynamic model for pipelines to establish the upper limit of water content in supercritical CO_2_ transport systems. The experimental results demonstrated that the critical relative humidity ranged from 50% to 60%. At a critical relative humidity of 50%, the corrosion rate of X70 steel was 0.0387 mm/year, with the primary corrosion products being FeSO_4_ crystalline hydrate and FeSO_3_ crystalline hydrate (Figure 3a). Liu et al. [39] investigated the corrosion behavior of X52 steel in a supercritical CO_2_ system containing multiple impurities, including O_2_, H_2_S, SO_2_, and NO_2_. The results revealed that, as the water content increased from 20 ppmv to 4333 ppmv, the corrosion rate of X52 steel correspondingly escalated from 0.0199 mm/y to 0.2838 mm/y (Figure 3b). Moreover, as the water content increases, the corrosion product film progressively transitions from predominantly FeOOH to a mixed film of FeSO_4_ and FeOOH. The critical water content that markedly alters the corrosion rate was 100 ppmv. Sun et al. [40] determined that the critical water content for X65 steel is 1500 ppmv when corroded in a supercritical CO_2_-H_2_O-O_2_-H_2_S-SO_2_ environment at 10 MPa and 50 °C. When the water content was below 1500 ppmv, the corrosion effect of impurity interactions dominated the corrosion process (Figure 3c). When the water content exceeded 1500 ppmv, the corrosion rate increased significantly, and the corrosion products included FeSO_4_, FeOOH, S, FeS_0.9_, and FeSO_3_. The corrosion process was governed by both the interactions between impurities and the corrosive effects of individual impurities.

**Figure 4:**
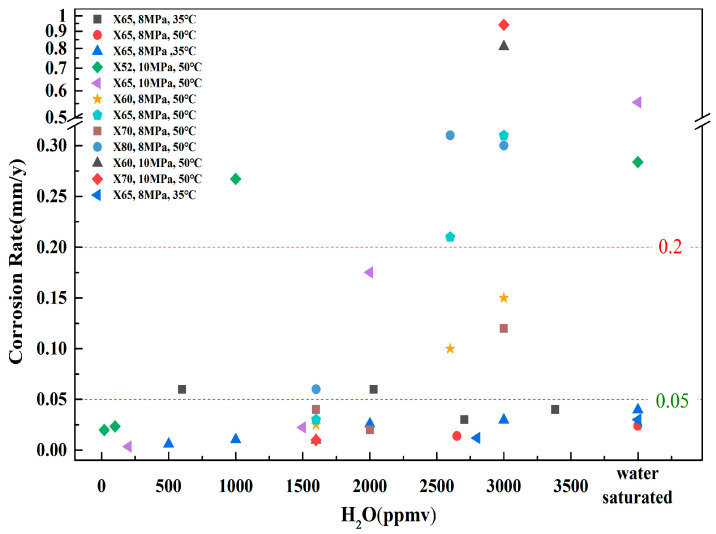
Corrosion rates for pipeline steels exposed to various water contents [35,36,37,39,40,41].

Currently, in CO_2_ transportation pipelines, API pipeline steels are predominantly utilized. Figure 4 summarizes average corrosion rates for five pipeline steels exposed to various water contents [35,36,37,39,40,41]. It is seen from the above studies that the influence of water content on the corrosion behavior of steel in a supercritical CO_2_ environment exhibits a critical threshold. The critical threshold varies depending on the material and corrosion environment. As the water content exceeds the critical value, a free water phase forms, leading to the development of a water film on the steel surface and the dissolution of CO_2_ and other impurities, which results in an increased rate of corrosion. Provided that the water content in supercritical CO_2_ remains below the critical threshold, the corrosion rate remains relatively low, irrespective of the presence of impurities [35,40,41,42,43,44]. It is noteworthy that, as water content varies, the local corrosion rate exhibits more rapid fluctuations. Moreover, the critical threshold of water content for local corrosion is significantly lower than that corresponding to the average corrosion rate under identical conditions [36,37,41,42,43]. As per the NACE standard RP0775-2023 [45], the corrosion behavior of carbon steel can be categorized into three distinct types: low corrosion (average general corrosion rate < 0.05 mm/y, maximum pitting rate < 0.13 mm/y), moderate corrosion (average general corrosion rate: 0.05–0.2 mm/y, maximum pitting rate: 0.13–0.3 mm/y), and high corrosion (average general corrosion rate > 0.2 mm/y, maximum pitting rate > 0.3 mm/y). In accordance with this standard, to ensure that the pipe material maintains a low corrosion level, it is essential to control the water content. It is evident from the data presented in Table 1 that, under identical conditions, when the maximum pitting rate is used as the criterion for evaluation, the permitted water content is significantly lower than that associated with the average general corrosion rate. Therefore, there is no universally accepted standard for the upper limit of water content in supercritical CO_2_.

### 3.2. O_2_ Content

When O_2_ is present in the corrosion system, it reacts with Fe^2+^ to progressively oxidize Fe^2+^ to Fe^3+^, leading to the formation of Fe(OH)_3_ and Fe_2_O_3,_ as indicated in Equations (12) and (13). This reaction disrupts the originally dense protective layer of FeCO_3_ and accelerates the corrosion process [46,47]. The presence of O_2_ can result in a localized oxygen concentration difference, which may lead to more severe localized corrosion. Under high temperature and pressure conditions, the coexistence of CO_2_ and O_2_ can lead to severe localized corrosion on the inner wall of the pipeline [48,49]. In supercritical CO_2_ transport, O_2_ can alter the solubility of H_2_O in supercritical CO_2_, modify the reaction pathway, and function as an oxidizer, thereby further influencing the corrosion process [50]. However, certain studies have demonstrated that, within a specific concentration range, increasing O_2_ concentration can actually decrease the corrosion rate. Table 2 presents a comprehensive summary of numerous authors’ results after conducing experiments in supercritical CO_2_ environments with differing O_2_ content levels.

(12) $${4Fe\left(OH\right)}_{2}+2H_{2}O+O_{2}\to 4Fe{\left(OH\right)}_{3}$$

(13) $$4Fe{\left(OH\right)}_{3}\to {Fe}_{2}O_{3}+3H_{2}O$$

**Figure 5:**
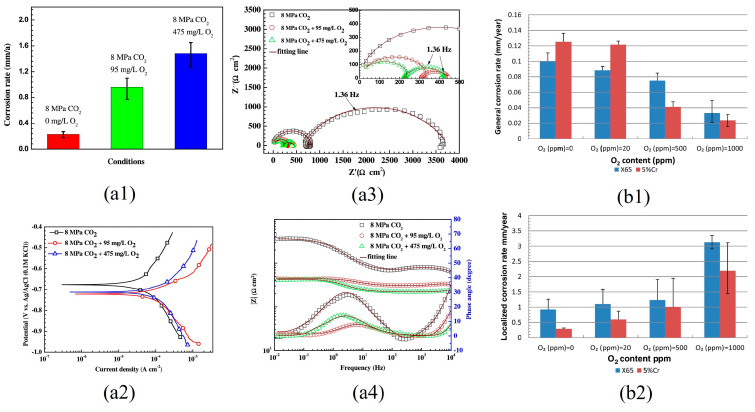
(**a1**) Corrosion rate of X65 carbon steel in water-saturated SC-CO_2_ phase without and with O_2_ for 96 h; (**a2**) Polarization curves of X65 carbon steel after corrosion in supercritical water-saturated CO_2_ phase without and with O_2_ for 96 h; (**a3**) EIS Nyquist and (**a4**) Bode plots (measured at open circuit potential) of X65 carbon steel in supercritical Water-saturated CO_2_ phase without and with O_2_ in the initial stage [50]; (**b1**) General corrosion rates of X65 carbon steel and 5Cr in supercritical water-saturated dense phase CO_2_ at 80 bar and 35 ◦C for an exposure time of 48 h at different O_2_ content (0, 20, 500 and 1000 ppm) and (**b2**) Local corrosion rates of X65 carbon steel and 5Cr in supercritical water-saturated CO_2_ phase at 80 bar and 35 °C for an exposure time of 48 h at different O_2_ content (0, 20, 500 and 1000 ppm) [42].

**Figure 6:**
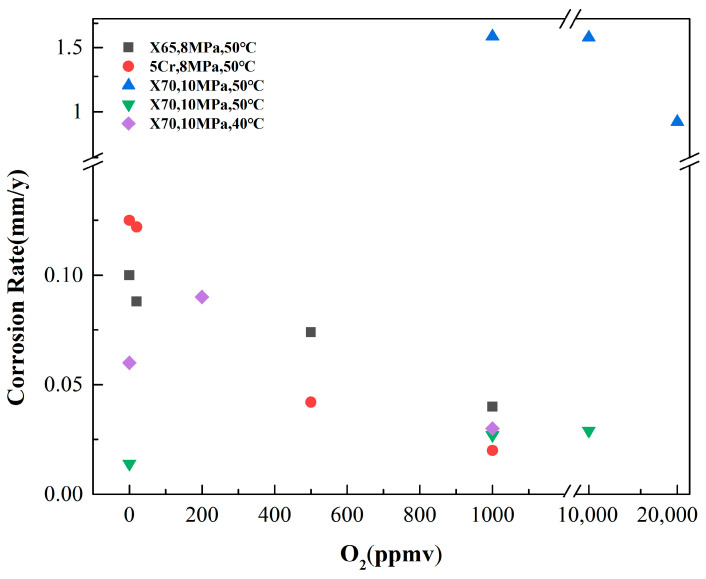
Corrosion rates for pipeline steels exposed to various O_2_ content [42,43,47,51].

Li et al. [32] investigated the corrosion behavior of various pipeline steels in a water-saturated supercritical CO_2_ environment containing 3% and 6% O_2_. The results revealed that, at pressures ≤ 9 MPa, the corrosion rates of several materials exhibited a slight increase with higher O_2_ concentrations. Sun et al. [47] also found that, within an O_2_ concentration range of 0–1000 ppm, the corrosion rate of X70 steel in supercritical CO_2_ environments increased progressively with rising O_2_ concentration. Electrochemical tests of X65 steel in supercritical CO_2_ environments with varying concentrations of O_2_ (95–475 mg/L) demonstrated that the addition of even small amounts of O_2_ caused a negative shift in the corrosion potential, an increase in corrosion current density, and a decrease in impedance [48]. These findings indicate that O_2_ significantly accelerates the corrosion of steel in these conditions. Further studies revealed that a minor amount of O_2_ inhibits the formation of the FeCO_3_ film, while promoting the development of porous Fe_2_O_3_. Additionally, as the concentration of O_2_ increases, the corrosion rate also rises (Figure 5(a1–a4)) [50]. However, Hua et al. [42] discovered that the impact of O_2_ content in water-saturated supercritical CO_2_ on uniform corrosion differs from its effect on local corrosion. The uniform corrosion rate of X65 and 5Cr steel decreased with the increase in O_2_ content, while the local corrosion rate increased. (Figure 5(b1,b2)). The presence of O_2_ facilitated the formation of Fe_2_O_3_ along with other oxides and hydroxides. While the thin amorphous oxide layer serves to mitigate uniform corrosion, the galvanic effect arising from the film’s heterogeneity exacerbates localized corrosion. Li et al. [49] s investigated the corrosion of N80 carbon steel in supercritical CO_2_ under the partial pressure of 0–10 bar O_2_ and found that the corrosion rate decreased with increase in the partial pressure of O_2_. In the initial stage of corrosion, O_2_ facilitated the formation of iron hydroxide and oxide films on the sample surface, creating a diffusion barrier layer. This led to the accumulation of Fe^2+^ and CO_3_^2−^, which subsequently resulted in the formation of a protective FeCO_3_ film that inhibited further steel corrosion. Xu et al. [43] investigated the corrosion behavior of X70 steel in a supercritical CO_2_-H_2_O-SO_2_-O_2_ system at 10 MPa and 50 °C, and found that the effect of O_2_ on corrosion rate varied with water content in dynamic condition. Specifically, within the relative water content range of 50–60%, the uniform corrosion of X70 steel increased as O_2_ concentration rose from 0.1 mol% to 1.0 mol%, and both the local corrosion rate and severity showed a significant increase with rising O_2_ concentration (from 0.1 mol% to 1.0 mol%) within the relative water content range of 50–88%. The critical relative water content was 60% when O_2_ content was 0.1 mol%, while the critical relative water content dropped to 45% when O_2_ content was 1.0 mol%. Wang et al. [52] found that the integrity of the FeCO_3_ corrosion product film was destroyed by low concentrations of O_2_, thereby accelerating corrosion in a water-saturated CO_2_ system. However, when the O_2_ concentration reached 1000 ppm, the steel matrix entered a passivation zone, leading to a decrease in the corrosion rate. Figure 6 summarizes average corrosion rates for three pipeline steels exposed to various O_2_ contents [42,43,47,51].

As previously discussed, the results in the published literature regarding the impact of O_2_ content on steel corrosion in a supercritical CO_2_ environment are inconsistent [32,42,43,47,48,49,50,51]. However, as the O_2_ content increases, the growth rate of the corrosion rate of steel exhibits a decreasing trend. In a supercritical CO_2_ environment, the presence of O2 may lead to the formation of iron oxides, such as Fe_2_O_3_, Fe_3_O_4_ and FeOOH. The alteration of the corrosion product film could influence the uniform corrosion process of steel. When the oxide film is relatively dense, it can inhibit the penetration of corrosive media, thereby mitigating the uniform corrosion of steel. The impact of O_2_ on the local corrosion rate is highly significant [42,43]. When the average corrosion rate decreases under conditions of high O_2_ content, the local corrosion rate may increase even further [42]. Numerous studies have demonstrated that O_2_ exhibits enhanced synergistic effects with other impurities in the system, such as H_2_O and SO_2_, thereby significantly influencing the corrosion process of steel [32,47,48,49]. Therefore, it is imperative to control the O_2_ content in transportation systems as much as possible. According to the current specifications of the international DYNAMIS project, the O_2_ concentration should be limited to no more than 1000 ppmv during enhanced oil recovery (EOR) applications [52].

### 3.3. H_2_S Content

If a condensed water film was formed on the pipeline steel, the dissolution of H_2_S in the water film and the subsequent dissociation would generate S^2−^, HS^−^ and H+, and the iron cations (Fe^2+^) could react with the anions (S^2−^) to form the iron sulfide layer on the surface of steel, as illustrated in Equations (14) [53] and (15) [54]. The deposition of these sulfides on the steel surface can exacerbate corrosion, leading to a significant reduction in the strength and toughness of the steel [53,54,55]. The presence of H_2_S not only affects the solubility of other impurities but also reduces the solubility of H_2_O in a CO_2_-H_2_S system compared to a pure CO_2_ system [56]. Table 3 presents a comprehensive summary of numerous authors’ results after experiments in supercritical CO_2_ environments with differing H_2_S content levels.

(14) $${Fe}^{2+}+S^{2-}\to FeS$$

(15) $${Fe}^{2+}+{HS}^{-}\to FeS+H^{+}$$

**Figure 7:**
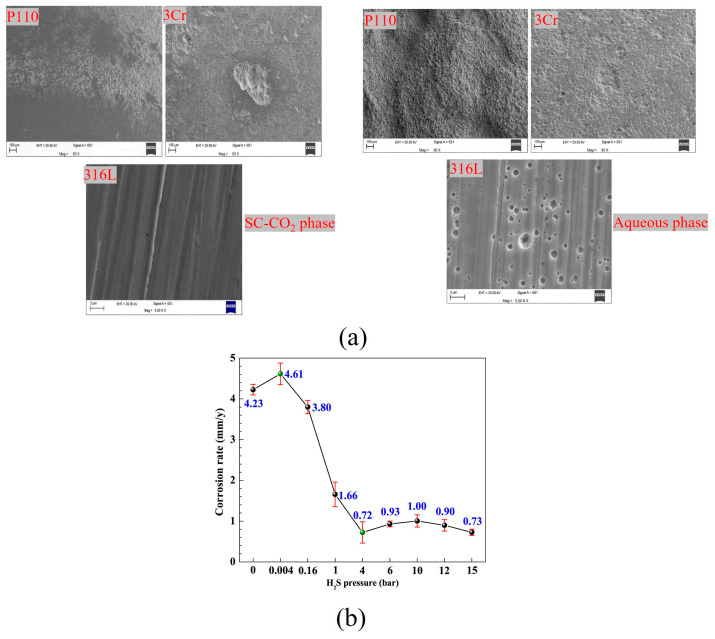
(**a**) SEM of three steels after scale removal [57]; (**b**) Corrosion rate of N80 steel was investigated under supercritical CO_2_-H_2_S environments at 80 bar and 80 °C for 72 h, with varying H_2_S pressures [54].

Sun et al. [54] discovered that N80 steel exhibits uniform corrosion in the absence of H_2_S; the corrosion rate of N80 initially increased slightly with the increase in H_2_S partial pressure, and then decreased significantly in a supercritical CO_2_ system containing H_2_S. When the partial pressure of H_2_S ranges from 0.004 bar to 4 bar, both uniform and localized corrosion occur. As the partial pressure of H_2_S increases further, uniform corrosion remains predominant, while the corrosion products progressively transform from FeCO_3_ to FeS (Figure 7b). They also found that the H_2_S-induced phase distribution changes point to the root cause of H_2_S-enhanced corrosion. Furthermore, preparing an amorphous Ni-P coating on X65 pipeline steel could prevent over 80% of localized corrosion [55]. Wei et al. [57] investigated the corrosion behavior of various steels in a dynamic supercritical CO_2_ environment with a lower H_2_S content (50 ppm). After 240 h of corrosion testing under dynamic conditions at 80 °C and 10 MPa, low alloy steel exhibited severe uniform and localized corrosion in an aqueous medium. In contrast, 316L stainless steel primarily experienced pitting corrosion. In supercritical CO_2_ environments, low alloy steel showed predominantly localized corrosion, whereas 316L stainless steel demonstrated strong corrosion resistance with no significant signs of corrosion (Figure 7a). They found that the presence of a small amount of H_2_S altered the adsorption behavior of H_2_O on the steel surface in a dynamic supercritical environment, leading to more extensive H_2_O adsorption across the entire surface, and accelerated both uniform and localized corrosion of X65 steel in the dynamic supercritical CO_2_ environment. The small amount of H_2_S played a more significant role in the dynamic CO_2_-saturated water phase, which changed the microstructure and composition of the corrosion products, and changed the dominated corrosion type of X65 steel from local corrosion to uniform corrosion [58]. Wang et al. [59] found that the corrosion rate of Q125 steel in supercritical CO_2_ increased significantly with the rise in H_2_S partial pressure, leading to more severe localized corrosion. The corrosion products evolved from a single-layer structure into a double-layer structure, with the inner layer comprising iron sulfide, FeCO_3_, and a minor amount of Cr(OH)_3,_ while the outer layer consisted primarily of FeCO_3_. Conversely, in the formation water phase, the corrosion rate decreased with increasing H_2_S partial pressure, and the corrosion products also exhibited a double-layer structure. It is evident that H_2_S alters the morphological structure of corrosion products, yet its influence on the corrosion rate varies depending on the material and corrosion system.

Through the aforementioned research, it can be conclusively stated that the presence of H_2_S in a supercritical CO_2_ environment accelerates the corrosion rate of steel. The corrosion products exhibit a distinct double-layered structure. Moreover, the influence of H_2_S on the corrosion behavior of steel in the water phase is significantly greater than that in the CO_2_ phase. H_2_S dissolves in water to form an acidic solution, which substantially increases the corrosion rate of steel. However, H_2_S does not accelerate the localized corrosion rate of steel. Furthermore, the presence of H_2_S in the water phase induces a transition in the corrosion behavior of steel from localized to general corrosion.

### 3.4. SO_2_ Content

In supercritical CO_2_ pipeline transportation, if there are H_2_O and SO_2_, SO_2_ dissolves in water to form H^+^ and HSO_3_^−^, which reduces the pH value of the system and accelerates the corrosion of the pipeline. In the CO_2_-SO_2_ system, the predominant corrosion product is precipitated FeSO_3_, where SO_2_ acts as the primary driving force [61,62]. In the CO_2_-SO_2_-O_2_ system, the presence of O_2_ causes the corrosion product FeSO_3_ to undergo further oxidation, resulting in the formation of FeSO_4_. If oxygen is present in sufficient quantities, FeOOH will be further oxidized, as illustrated in Equations (16)–(20) [63,64]. Table 4 presents a comprehensive summary of numerous authors’ results after conducting experiments in supercritical CO_2_ environments with differing SO_2_ content levels.

(16) $$H_{2}O+{SO}_{2}\to H^{+}+{{HSO}_{3}}^{2-}$$

(17) $${{HSO}_{3}}^{2-}\to H^{+}+{{SO}_{3}}^{2-}$$

(18) $${{SO}_{3}}^{2-}+{Fe}^{2+}\to {FeSO}_{3}$$

(19) $${Fe}^{2+}+{{SO}_{4}}^{2-}\to {FeSO}_{4}$$

(20) $$4{FeSO}_{4}+6H_{2}O+O_{2}\to 4FeOOH+4H_{2}{SO}_{4}$$

The corrosion rate of X70 steel was observed to increase as the SO_2_ concentration rose in a dynamic supercritical environment. The primary corrosion products identified were FeSO_4_ and FeSO_3_·XH_2_O, which formed a protective layer on the specimen surface, thereby reducing the corrosion rate [61]. Hua et al. [62] investigated the corrosion behavior of X65 steel in both water-saturated and unsaturated supercritical CO_2_ environments and found that the presence of O_2_ and SO_2_ impurities significantly increased the corrosion rate, leading to pronounced pitting corrosion. In the absence of O_2_ and SO_2_, the critical water content required to maintain an overall corrosion rate below 0.1 mm/year was 3400 ppm, which was close to the solubility limit of water in CO_2_ under the corresponding conditions. However, with the addition of 50 ppm SO_2_ and 20 ppm O_2_, the critical water content decreased to 2120 ppm. When the SO_2_ concentration was increased to 100 ppm, the critical water content further reduced to 1850 ppm. Notably, the critical water content required to mitigate localized corrosion was 500 ppm, regardless of the SO_2_ concentration.

**Figure 8:**
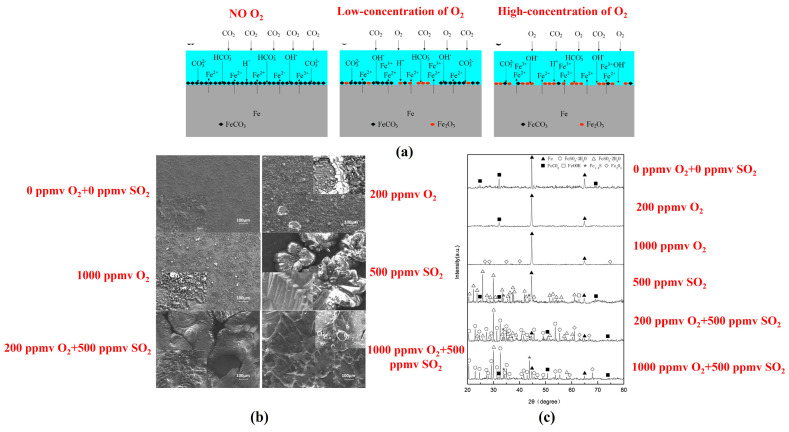
(**a**) Schematic diagram of the corrosion models of X70 steel in water-saturated supercritical CO_2_; (**b**) SEM surface morphologies of corroded X70 steel in water-saturated supercritical CO_2_ with different gas impurities; and (**c**) XRD results of X70 steel in water-saturated supercritical CO_2_ with different gas impurities (40 °C, 100 bar) [51].

Mahlobo et al. [66] investigated the corrosion behavior of pipeline steel in a supercritical CO_2_ environment containing 0.5–5% SO_2_ impurities over a period of 1512 h. Their results demonstrated that, as the SO_2_ concentration increased to 5%, the corrosion rate markedly escalated, reaching a peak of 1.396 mm/year. Given that the solubility of SO_2_ is higher than that of CO_2_, the SO_2_ system at high concentrations exhibits greater corrosiveness. Wang et al. [51] investigated synergistic effect of O_2_ and SO_2_ gas impurities on X70 steel corrosion in water-saturated supercritical CO_2_ environment and found the impact of SO_2_ was more pronounced than that of O_2_. The synergistic effect between SO_2_ and O_2_ was influenced by their varying concentrations; higher concentrations of O_2_ exhibited a certain inhibitory effect on the corrosion process (Figure 8). Sun et al. [60,64] found that, in water-saturated supercritical CO_2_ systems, SO_2_ exerted a more pronounced effect on the average corrosion rate of X65 steel compared to H_2_S and O_2_ when present as single impurities. The synergistic effects of multiple impurities on corrosion rates were observed in the following order: S_O2-H2S_ (4.88), S_O2-SO2_ (35.69), S_H2S-SO2_ (42.03), and S_O2-H2S-SO2_ (54.88). In the presence of both H_2_S and SO_2_, sulfur can be produced, which subsequently reacts with water to form sulfuric acid (H_2_SO_4_), as shown by Equations (21) and (22):

(21) $${2H}_{2}S+{SO}_{2}\to 3S+2H_{2}O$$

(22) $$8S+8H_{2}O\to {6H}_{2}S+2H_{2}SO_{4}$$

Similar to the effect of H_2_S, the presence of SO_2_ accelerates the corrosion rate of steel in supercritical CO_2_ environments. The composition and morphology of corrosion products are highly complex, exhibiting a multi-layered structure. When SO_2_ is present as a sole impurity gas, it does not significantly influence the localized corrosion behavior of steel in a supercritical CO_2_ environment.

### 3.5. NO_2_ Content

NO_2_ is a potent oxidizing gas that readily dissolves in water to form nitric acid (HNO_3_), thereby reducing the pH of the system. This has a significant impact on corrosion behavior in supercritical CO_2_ environments. Corrosion products may include oxides and nitrates, such as Fe(NO_3_)_3_, as indicated in Equations (23)–(25) [67]. These products can dissolve the surface protective layer, thus altering corrosion behavior and potentially leading to pitting corrosion at higher concentrations [34]. Table 5 presents a summary of the experimental results obtained in a supercritical CO_2_ environments contaminated with NO_2_ impurities.

(23) $$3{NO}_{2}+H_{2}O\to NO+2{HNO}_{3}$$

(24) $${Fe}^{3+}+3{{NO}_{3}}^{-}\to {Fe\left({NO}_{3}\right)}_{3}$$

(25) $$4{Fe\left({NO}_{3}\right)}_{3}\to 2{Fe}_{2}O_{3}+12{NO}_{2}+3O_{2}$$

**Figure 9:**
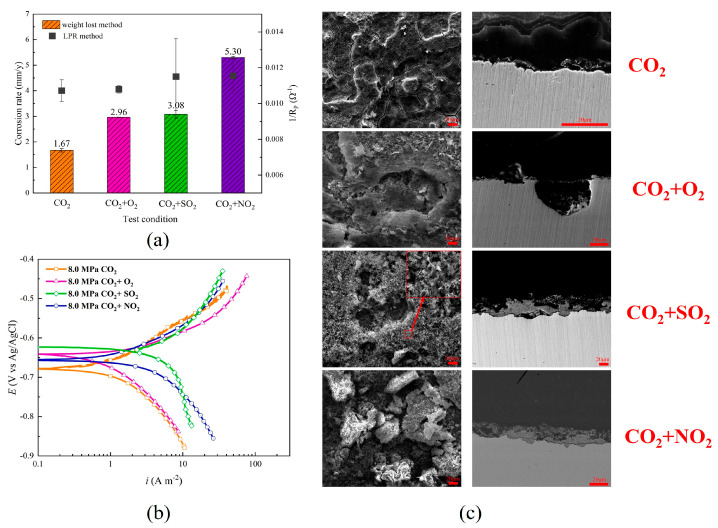
(**a**) General corrosion rate and 1/R_p_ of X80 steel immersed in water-rich phases containing O_2_, SO_2_ or NO_2_ at 8 MPa and 35 °C for 48 h; (**b**) Polarization curve of X80 steel immersed in water-rich phases containing O_2_, SO_2_ or NO_2_ at 8 MPa and 35 °C for 48 h; and (**c**) SEM surface and cross-sectional morphology of X80 steel immersed in the water-rich phase containing O_2_, SO_2_ or NO_2_ at 8 MPa and 35 °C for 48 h [63].

Morland et al. [67] discovered that, in dense phase CO_2_ at 25 °C and 10 MPa, carbon steel began to corrode when the NO_2_ content was 75 ppmv and the water content reached 350 ppmv. The corrosion significantly intensified when the water content increased to 670 ppmv, with a corrosion rate of 0.57 mm/year. The corrosion products transitioned from black to brown, indicating the formation of iron oxides, though the specific type remains undetermined. Given the conditions of the corrosive medium, it is plausible that carbon steel may form FeOOH upon exposure to NO, NO_2_, or HNO_3_. This observation was also corroborated by other researchers [63,68]. Li et al. [63] discovered that the corrosion rate of X80 steel was 5.30 mm/y after 48 h in a supercritical CO_2_ environment containing 100 ppmv of NO_2_, which was significantly higher than that in an environment with O_2_, SO_2_, and without impurities. HNO_3_ released H^+^ ions, thereby facilitating the cathodic reaction, and the corrosion products formed display inadequate adhesion to the steel matrix and insufficient protective properties, consequently accelerating the corrosion of X80 steel. (Figure 9). In dense phase CO_2_ environments containing NO_2_ and H_2_O, the corrosion rate of X65 steel ranged from 0.06 to 1.66 mm/year, which was significantly higher than that observed in similar concentrations of SO_2_ impurities [68]. Sun et al. [69] investigated the corrosion behavior of X65 steel in supercritical CO_2_ saturated with water containing O_2_, H_2_S, SO_2_, and NO_2_ impurities at 10 MPa and 50 °C. Their findings indicated that NO_2_ is the dominant factor controlling the corrosion behavior of X65 steel, and NO_2_ and SO_2_ exert the most significant influence on the corrosion of X65 steel, followed by H_2_S and O_2_. Local corrosion occurred in an environment containing only NO_2_ impurities, where the corrosion products primarily consisted of Fe_2_O_3_·H_2_O, with minor amounts of Fe(NO_3_)_3_·9H_2_O.

They also found that the influence of different impurities affects stress corrosion cracking (SCC) sensitivity. Specifically, O_2_ has minimal impact on enhancing SCC susceptibility of X65 steel, whereas SO_2_ and NO_2_ greatly enhance SCC susceptibility due to their strong corrosion effects. The SCC process of X65 steel in a supercritical CO_2_ environment also varies based on the type of impurities [70]. There is a relative paucity of studies focusing on NO_2_ as an individual impurity, with more research concentrating on the synergistic effects within multi-impurity systems.

There is a limited number of studies investigating the influence of NO_2_ on the corrosion behavior of steel in supercritical CO_2_ environments. Based on existing research findings, NO_2_ has been shown to accelerate steel corrosion in such environments and induce local corrosion. When present as a single impurity gas, NO_2_ exerts a greater impact on the corrosion rate of steel in supercritical CO_2_ environments compared to O_2_, SO_2_, and H_2_S.

**Table 5:** Corrosion behavior of steel with NO_2_ impurities in supercritical CO_2_.

Materials	Pressures (MPa)	Temperature (°C)	Environment	NO_2_ Content	Other Impurities	Corrosion Time (h)	Corrosion Rate (mm/y)	Corrosion Products	Reference
X80	8	35	CO_2_-saturated 1wt% NaCl solution	0		48	1.67	FeCO_3_, Fe_2_O_3_	[63]
				100 ppmv			5.30	FeCO_3_, FeOOH	
X65	10	50	water-saturated CO_2_ phase	0		24	~0.04	FeCO_3_	[69]
				1000 ppmv			1.72–1.76	FeCO_3_, Fe(NO_3_)_3_∙9H_2_O, Fe_2_O_3_∙H_2_O	
				0		120	<0.04	FeCO_3_	
				1000 ppmv			0.44–0.48	FeCO_3_, Fe(NO_3_)_3_∙9H_2_O, Fe_2_O_3_∙H_2_O	

### 3.6. Influence of Working Conditions (Temperature, Pressure, Flow Rate, Time)

Changes in temperature and pressure not only influence the solubility of H_2_O in supercritical CO_2_ and the solubility of impurity gases in CO_2_ within H_2_O, but also alter the morphology of corrosion products and the reaction rate [71].

**Figure 10:**
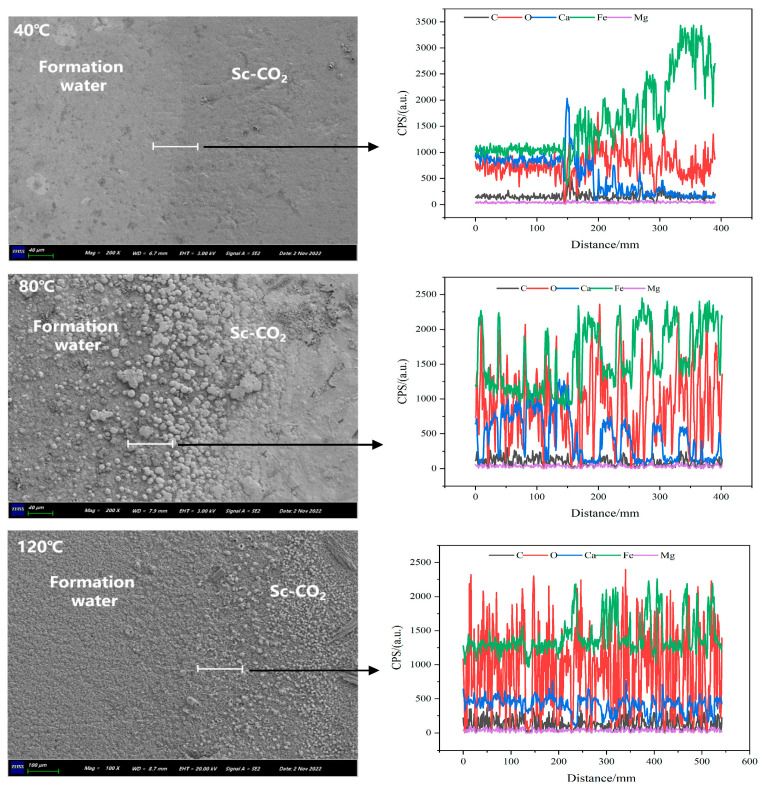
Distribution of the key elements at the surfaces of N80 steel samples determined by means of EDS linear scanning (along the white lines) after immersion experiments at 40 °C, 80 °C and 120 °C for 7 days [75].

Nazari et al. [72] discovered that the morphology and stability of corrosion products on X70 steel are significantly influenced by temperature in a CO_2_ environment ranging from 55 °C to 85 °C. At higher temperatures, the corrosion products become denser and more resistant to disintegration or detachment, thereby effectively inhibiting further corrosion of the substrate and enhancing its protective efficacy. Sui et al. [73] investigated the corrosion behavior of X65 steel in a supercritical CO_2_ environment across various temperatures and pressures. Their findings revealed that the most severe surface corrosion occurred at 8 MPa and 35 °C, with a corrosion rate of 0.190 mm/year. When the temperature increased to 50 °C, the corrosion rate decreased to 0.032 mm/year. Likewise, at a constant temperature of 35 °C, as the pressure was raised to 10 MPa, the corrosion rate reduced to 0.073 mm/year. Hua et al. [74] investigated the corrosion behavior of X65 steel in supercritical CO_2_ environments at varying temperatures and water contents. Despite similar molar concentrations of water at both temperatures, the CO_2_ density difference results in more than double the mass of water being dissolved into the CO_2_ phase per unit volume at 35 °C compared to 50 °C. This leads to a higher corrosion rate at 35 °C relative to 50 °C under water-saturated CO_2_ conditions. Additionally, the corrosion product film formed at 50 °C was denser and more protective. Wang et al. [75] investigated the corrosion behavior of N80 steel under experimental conditions involving formation water and supercritical CO_2_ at pressures of 10 MPa and temperatures ranging from 40 °C to 120 °C. They discovered that increasing temperature facilitated the formation of FeCO_3_. In both environments, the corrosion rate of N80 initially increased with rising temperature but subsequently decreased at higher temperatures. However, the corrosion products formed in different environments exhibit markedly distinct characteristics. In a formation water environment, the corrosion products tend to be more uniform and regular. In contrast, those formed in a supercritical CO_2_ environment display a variety of morphologies, including spherical, lamellar, and needle-like structures (Figure 10).

The influence of pressure on solubility leads to a decrease in the pH of the corrosive medium, an increase in the activity of corrosive ions, and alterations in the electric double layer at the steel/liquid interface. Consequently, in both O_2_-free and O_2_-containing water-saturated CO_2_ systems, the corrosion rates of several pipeline steels increased as pressure rose from 6.2 to 9 MPa, with a more rapid increase observed at 10 MPa [32]. Xu et al. [41] studied the corrosion behavior of supercritical CO_2_ containing SO_2_ and O_2_ by water content on several pipeline steels at 50 °C and 8–12 MPa. The research revealed that pressure significantly influences the solubility of water in CO_2_, thereby affecting the corrosion rate. Specifically, when the water content was below 2600 ppm, the corrosion rate at 10 MPa was marginally lower than that at 8 MPa. However, with a water content of 3000 ppm, the corrosion rate at 10 MPa markedly increased, surpassing the rate observed at 8 MPa. Additionally, the surface corrosion morphology of the X65 steel sample exhibited changes under these conditions. In the study of CO_2_-saturated water phase, Rodrigues et al. [76] investigated the effect of pressure on CO_2_ corrosion when water was the sole impurity at 50 °C and at pressures ranging from 2 to 20 MPa. FeCO_3_ scales formed under supercritical pressures and, in CO_2_-saturated water, exhibited significantly enhanced protective properties. Compared to the corrosion rate in subcritical wet CO_2_ environments (0.05–0.09 mm/year), the corrosion rate in the CO_2_-saturated water phase showed a significant increase, reaching 0.59–5.09 mm/year.

**Figure 11:**
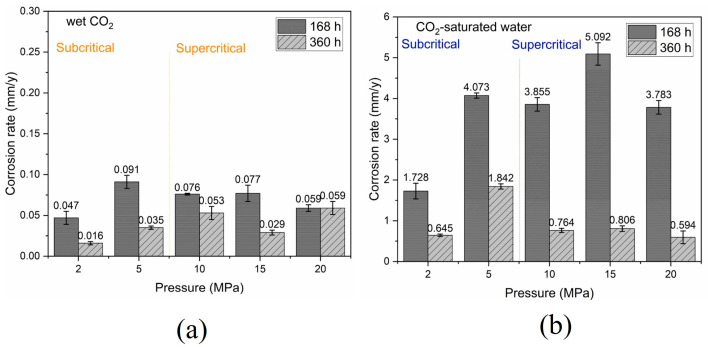
General corrosion rate of steel after exposure to CO_2_ medium at subcritical and supercritical pressures (**a**) wet CO_2_; (**b**) CO_2_-saturated water [76].

The flow rate increases the accessibility of corrosive ions (H^+^) to the surface of the metal matrix, thereby accelerating the corrosion process. Additionally, it carries Fe^2+^ ions away from the matrix surface, which hinders the formation of the protective FeCO_3_ film [77]. Wei et al. [78] investigated the corrosion behavior of X70 steel in CO_2_-saturated water under both static and dynamic conditions (0–2 m/s) and obtained consistent conclusions. The corrosion rate and pit depth were significantly higher under dynamic conditions compared to static conditions, suggesting that localized corrosion predominates when fluid motion is present (Figure 11). They also discovered that the variation in flow rate altered the predominant corrosion type in the supercritical CO_2_-saturated water phase from uniform corrosion to localized corrosion. The number and size of FeCO_3_ particles increased over time, leading to a thicker and denser FeCO_3_ corrosion product layer, which in turn enhanced the protective effect on the substrate. However, under dynamic conditions during the initial stage of corrosion, the absence of corrosion products allows the corrosive medium to easily reach the substrate surface, resulting in severe localized corrosion [78]. Zeng et al. [79] discovered that, in a static supercritical CO_2_ environment, uniform corrosion predominates whereas, under dynamic conditions, localized corrosion is more pronounced and the corrosion rate increased significantly. Li et al. [80] found that the increase in flow velocity (from 0 to 2 m/s) disrupts the densification of the FeCO_3_ corrosion product film, thereby diminishing its protective effect on the substrate and accelerating the corrosion rate. Electrochemical test results also indicate that, as flow velocity increases, the self-corrosion current density rises while impedance decreases. Wang et al. [81] investigated the corrosion behavior of 13Cr SS in the presence of CO_2_ and H_2_S at flow rates ranging from 0 to 2.5 m/s. They discovered that the corrosion product film was more prone to detachment in the static water phase compared to the CO_2_ phase. Consequently, after 336 h, the thickness of the corrosion product layer on the sample surface in the aqueous phase was significantly lower.

The corrosion duration in a supercritical CO_2_ environment not only influences the thickness and composition of the corrosion product film but also impacts its protective properties, the initiation of localized corrosion, and changes in the corrosion rate. Mahlobo et al. [66] investigated the long-term corrosion behavior of carbon steel in supercritical CO_2_ environments and found that the protective efficacy of the corrosion product film increased progressively over time, leading to a lower corrosion rate after 1512 h compared to that observed at 336 h. Wang et al. [82] proposed that, in the H_2_O-rich phase, as corrosion time increased, the corrosion product layer evolved from porous to dense, ultimately forming a protective layer. In the CO_2_-rich phase, corrosion products predominantly formed in water condensation regions, resulting in the formation of FeCO_3_. Hua et al. [36] discovered that, in the CO_2_-saturated water phase, the types and forms of corrosion products evolved over time. In comparison with the CO_2_-saturated water phase, uniform corrosion is more prevalent in environments where water is less abundant. They also found that, as the exposure time of X65 and 13Cr in water-saturated supercritical CO_2_ containing SO_2_ and O_2_ increased from 6 h to 48 h, the uniform corrosion rate and local corrosion rate decreased, but the local corrosion depth increased [83].

Compared with the influence of pressure, the conclusions drawn from various studies regarding the effects of temperature, flow rate, and time on the corrosion behavior of steel in supercritical CO_2_ environments are relatively consistent. Temperature variations can significantly influence the morphology of corrosion products and modulate the rate of corrosion reactions. Overall, at higher temperatures, the corrosion products formed on steel in a supercritical CO_2_ environment are denser, which enhances the protective barrier for the substrate and consequently reduces the corrosion rate. The composition and morphology of the corrosion products evolve over time. As time progresses, the corrosion products will gradually thicken and densify, consequently reducing the corrosion rate of the steel. In a supercritical CO_2_ environment, the flow of fluids can erode corrosion products on the steel surface, potentially causing their detachment and thereby diminishing their protective capabilities. Additionally, the flow process enhances the transport of corrosive ions, such as H^+^, HCO_3_^−^, and CO_3_^2−^, thereby accelerating both the average and localized corrosion rates of steel. Pressure not only affects the solubility of CO_2_ and various impurity gases in water but also influences the solubility of impurities in CO_2_. Additionally, it can alter the morphology of corrosion products, thereby exerting a complex influence on the corrosion behavior of steel in supercritical CO_2_ environment. For different corrosive environments and materials, the variation trend in the corrosion rate of steel with pressure does not follow a consistent pattern.

## 4. Corrosion of Welded Joints in Supercritical CO_2_ Environments

Welded joints are among the most susceptible components in pipeline systems. During the welding process, these joints experience a welding heat cycle, with heating and cooling occurring in an extremely non-uniform manner across different regions. The microstructural changes in the weld and heat-affected zones, along with the heterogeneity of welding materials, contribute to the vulnerability of these joints to corrosion [84,85,86,87].

**Figure 12:**
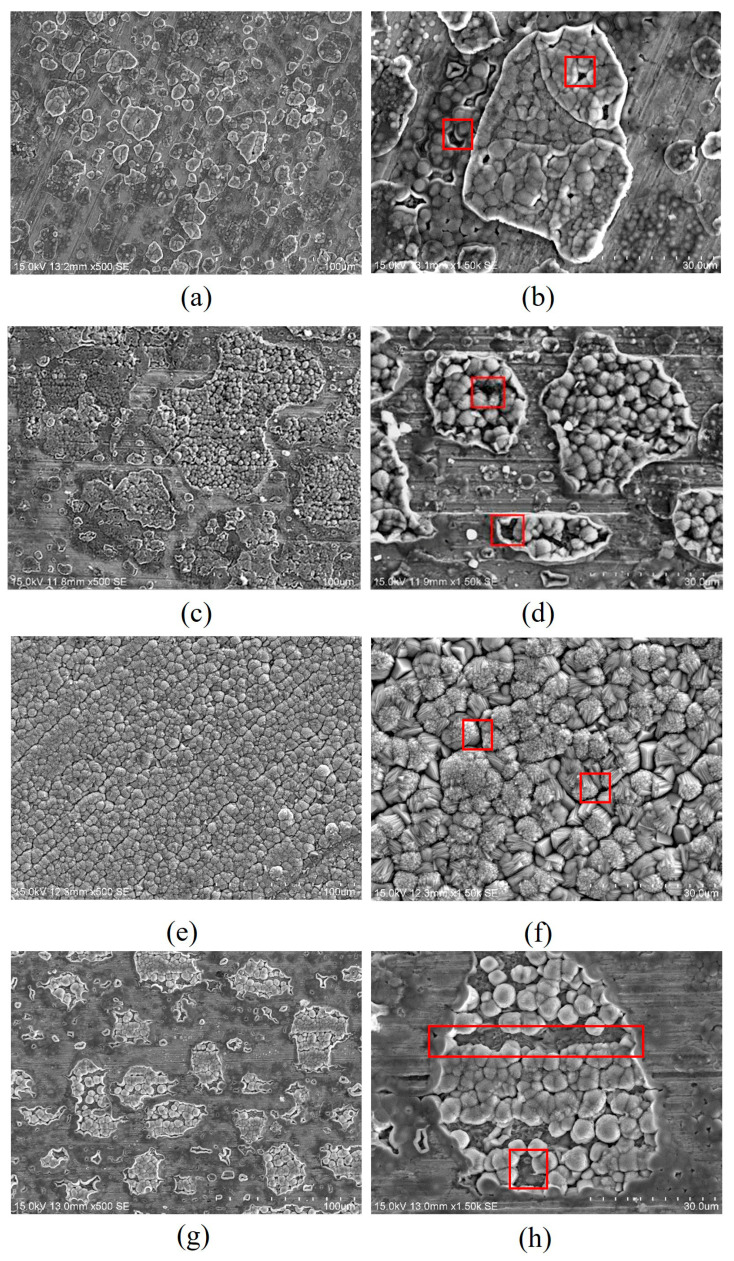
SEM surface morphologies of the X80 welded joint after corrosion in a water-saturated supercritical CO_2_ phase for 120 h: (**a**,**b**) BM; (**c**,**d**) FGHAZ; (**e**,**f**) CGHAZ; and (**g**,**h**) WM [91].

Ding et al. [88] investigated the corrosion mechanism in different regions of the X 65 welded joints in CO_2_/SO_2_ saturated aqueous solution. They found that SO_2_ and its hydrates were more inclined to adsorb in the BM and generate FeS product films, while the WM exhibited the poorest adsorption. This can be attributed to the differences in microstructure between the base material (BM), heat-affected zones (HAZ), and weld metal (WM), which result in varying formation of corrosion product films on the surfaces. Bai et al. [89] argue that welds and heat-affected zones (HAZs) are the most susceptible areas to failure in welded joints within the pipeline transportation of the oil and gas industry. Given that welding is inevitable in supercritical CO_2_ long-distance transportation pipelines, the corrosion behavior and protective measures for welded joints in supercritical CO_2_ environments become particularly critical. Regarding the research on welded joints, Lu et al. [90] investigated the early corrosion behavior of carbon steel welded joints in water-saturated CO_2_ oil fields. They discovered that the early corrosion characteristics of different regions within the welded joints were influenced by grain orientation but not by grain size, grain boundary type, or secondary phases. Yan et al. [91] investigated the corrosion behavior of X80 welded joints under conditions of 40 °C and 10 MPa water-saturated supercritical CO_2_. They found that the corrosion behavior varied due to differences in microstructure among the BM, fine-grained heat-affected zone (FGHAZ), coarse-grained heat-affected zone (CGHAZ), and WM. Specifically, FGHAZ and CGHAZ exhibited higher proportions of pearlite and more severe corrosion, while BM and WM, which contained higher ferrite content, experienced relatively mild corrosion. As shown in Figure 12, the surfaces of these different regions were covered with FeCO_3_ crystals, but the extent of coverage differed. The FeCO_3_ islands were larger on the surface of FGHAZ, whereas they were smaller but more numerous on the surfaces of BM and WM. CGHAZ showed the most severe corrosion, forming multilayer polycrystalline FeCO_3_ films that almost completely covered the specimen surface. There were numerous pores observed on both the FeCO_3_ islands on the sample surface in each zone, as indicated by the red frame, which could potentially serve as initiation sites for local corrosion. Yao et al. [92] also observed that, after 120 h of corrosion at 40 °C and 10 MPa, the corrosion levels of the X80 base metal and weld area were similar, with FGHAZ experiencing the least corrosion and CGHAZ showing the most severe corrosion. Additionally, the local corrosion depth of the joint was greater. The variation in chemical composition was identified as the primary factor responsible for the differing early corrosion behaviors across various regions of the welded joints.

Although research on welded joints in CO_2_ environments has reached a relatively mature stage, studies focusing on welded joints in supercritical CO_2_ conditions remain limited. The existing research primarily concentrates on the differences in corrosion product forms across various regions of the X80 welded joint, but lacks a detailed examination of corrosion rates and the associated corrosion mechanisms in welded joints. Currently, there is limited research available regarding the corrosion behavior of welded joints across different steel grades, as well as the influence of various types and concentrations of impurities on the welded joints in supercritical CO_2_ transmission pipelines.

## 5. Conclusions

The CCUS project is a large-scale system, and therefore investigating corrosion in supercritical CO_2_ pipeline transportation is crucial for ensuring operational safety.

1. When H_2_O is present in supercritical CO_2_ systems, gas impurities react with water to form corrosive products. Extensive studies have demonstrated that the corrosion rate increases significantly once the water content surpasses a specific critical threshold. However, the critical water content varies among different corrosion systems and materials. Under identical water content conditions, the localized corrosion rate can be several dozen times higher than the average corrosion rate. Controlling water content is crucial for mitigating pipeline corrosion. Compared to the average corrosion rate, taking the local corrosion rate into account is more reasonable for controlling water content in supercritical CO_2_ fluids. It is crucial to emphasize the impact of water content on the localized corrosion behavior of pipeline steel in future research.
2. A small amount of O_2_ accelerates the corrosion of steel; however, at high concentrations, O_2_ can induce passivation of the substrate, thereby significantly reducing the corrosion rate. O_2_ can facilitate local corrosion of steel in supercritical CO_2_ environments and significantly influence the rapid progression of localized corrosion. Acidic gases, such as H_2_S, SO_2_ and NO_2_, can accelerate the corrosion rate of steel, and their influence on the corrosion behavior of steel in the water phase is significantly greater than that in the CO_2_ phase. Among these gases, NO_2_ exhibits the most significant impact, often leading to localized corrosion. H_2_S and SO_2_ do not accelerate the localized corrosion rate of steel. When various types of gas impurities are present, the composition of corrosion products becomes more complex, and the corrosion products form a multi-layer structure. The synergistic effects of multiple impurities are more detrimental than those of individual impurities, and water content plays a crucial role in multi-impurity systems. The influence of the synergistic effect of multiple impurities on the corrosion behavior of pipeline steel requires further investigation and merits considerable attention.
3. Temperature and pressure significantly influence the formation and properties of corrosion product films. Numerous studies have demonstrated that, within a specific temperature range, elevated temperatures can promote the densification of corrosion product films, thereby enhancing the protective effect on the substrate surface. Moreover, the effects of pressure exhibit variability across different systems. The corrosion rate of steel increases with the flow velocity of supercritical CO_2_ fluid, and the dynamic flow of the fluid can lead to localized corrosion of steel. The morphology and structure of the corrosion products evolve as the corrosion time increases.
4. Research on welded joints of pipeline steel in the supercritical CO_2_ environment remains relatively limited. Existing studies indicate that distinct morphologies of corrosion products form in the HAZ, WM, and BM of X80 welded joints under supercritical CO_2_ conditions. However, a comprehensive and in-depth understanding of the corrosion mechanism remains insufficient. Specifically, there is a lack of comprehensive investigation into the influence of water content and impurity gases on the corrosion behavior of different pipeline steel welded joints.

## Figures and Tables

**Table 1:** Corrosion behavior of steel under different water content conditions.

Materials	Pressures (MPa)	Temperature (°C)	Water Content	Other Impurities	Test Period (h)	Corrosion Rate (mm/y)	Local Corrosion Rate (mm/y)	Reference
X65	8	35	2029 ppm (60% RH)		336	<0.02		[35]
					72	<0.1		
			2705 ppm (80% RH)					
			3382 ppm (water-saturated CO_2_ phase)					
			CO_2_-saturated water phase			1		
X65	8	50	700 ppm, 1600 ppm		48	No measurable corrosion	No measurable corrosion	[36]
			2650 ppm			0.014	0.2	
			3400 ppm (water-saturated CO_2_ phase)			0.024	1.4	
			300 mL (CO_2_-saturated water phase)		96	4.1		
X65	8	35	500 ppmv		72	0.0061	0.137	[37]
			1000 ppmv			0.0103	0.322	
			2000 ppmv			0.0259	0.697	
			3000 ppmv			0.0298	0.825	
			5000 ppmv			0.0398	1.127	
X70	10	50	0.95 g (50% RH)	SO_2_ 2.0 mol%, O_2_ 1000 ppm	120	0.0387		[38]
			1.32 g (70% RH)			0.3–0.4		
			1.67 g (88% RH)			0.8–0.9		
			3 g (100% RH)			1.4–1.5		
X52	10	50	20 ppmv	O_2_ 200 ppmv, H_2_S 200 ppmv, SO_2_ 200 ppmv	72	0.0199		[39]
			100 ppmv			0.0234		
			1000 ppmv			0.2671		
			4333 ppmv (water-saturated CO_2_ phase)			0.2838		
X65	10	50	200 ppmv	O_2_ 200 ppmv, H_2_S 200 ppmv, SO_2_ 200 ppmv	72	0.0036		[40]
			1500 ppmv			0.0224		
			2000 ppmv			0.1752		
			4333 ppmv (water-saturated CO_2_ phase)			0.5546		
X60, X65, X70, X80	8	50	1600 ppm	SO_2_ 3000 ppm, O_2_ 1000 ppm	72	0.025–0.06	0.2–3.25	[41]
			2600 ppm, 3000 ppm			0.1–0.31		
	10		1600 ppm, 2000 ppm, 2600 ppm			<0.01	0.04–6.02	
			3000 ppm			0.81–0.94		
X65	8	35	300 ppm, 650 ppm, 1200 ppm	O_2_ 1000 ppm	48	No measurable corrosion	No measurable corrosion	[42]
			2800 ppm			0.010–0.014	0.8	
			34,000 ppm (water-saturated CO_2_ phase)			0.03	3.1	
			34,000 ppm (water-saturated CO_2_ phase)			0.10	0.9	
5Cr	8	35	300 ppm, 650 ppm, 1200 ppm	O_2_ 1000 ppm	48	No measurable corrosion	No measurable corrosion	[42]
			2800 ppm			<0.01	0.7	
			34,000 ppm (water-saturated CO_2_ phase)			0.02	2.2	
			34,000 ppm (water-saturated CO_2_ phase)			0.125	0.3	
X70	10	50	45% RH	O_2_ 0.1 mol%	72	0.03	0.03	[43]
			60% RH			0.1		
			75% RH			0.90	~1.5	
			88% RH			~1.1	~3	
			100% RH			1.61	7.03	
			45% RH	O_2_ 1.0 mol%		0.03	0.03	
			50% RH			0.50		
			60% RH			0.63		
			75% RH			0.90		
			88% RH			0.90–1.0	9	
			100% RH			1.6	5.23	

**Table 2:** Corrosion behavior of steel with various O_2_ content in supercritical CO_2_.

Materials	Pressures (MPa)	Temperature (°C)	Environment	O_2_ Content	Other Impurities	Test Period (h)	Corrosion Rate (mm/y)	Local Corrosion Rate (mm/y)	Corrosion Products	Reference
X65	8	35	water-saturated CO_2_ phase	0 ppm		48	0.10	0.9	FeCO_3_, Fe_2_O_3_, FeOOH, Fe_3_O_4_	[42]
				20 ppm			0.088	1.0–1.2		
				500 ppm			0.072–0.076	1.2–1.3		
				1000 ppm			0.04	3		
5Cr	8	35	water-saturated CO_2_ phase	0 ppm		48	0.125	0.3	FeCO_3_, Fe_2_O_3_, Cr_2_O_3_, FeOOH, Cr(OH)_3_, Fe_3_O_4_	[42]
				20 ppm			0.120–0.124	0.5–0.6		
				500 ppm			0.040–0.044	1.0		
				1000 ppm			0.02	2.2		
X70	10	50	water-saturated CO_2_ phase	0.1 mol%	SO_2_ 2 mol%	72	1.61	7.03	FeO FeSO_4_∙xH_2_O	[43]
				1.0 mol%			1.6	5.23		
				2.0 mol%			0.94	3.3		
X70	10	50	water-saturated CO_2_ phase	0 ppm		120	0.014		FeCO_3_, Fe_2_O_3_	[47]
				1000 ppm			0.027			
				10,000 ppm			0.029			
N80	8	65	simulated formation water phase	0 bar		48	27.86		FeCO_3_, Fe_2_O_3_, FeOOH	[49]
				5 bar			13.15			
X65	8	50	water-saturated CO_2_ phase	0 mg/L		96	0.25		FeCO_3_, Fe_2_O_3_	[50]
				95 mg/L			0.91			
				475 mg/L			1.52			
X70	10	40	water-saturated CO_2_ phase	0		48	0.06		FeCO_3_	[51]
				200 ppmv			0.09			
				1000 ppmv			0.03		Fe_2_O_3_	

**Table 3:** Corrosion behavior of steel with various H_2_S content in supercritical CO_2_.

Materials	Pressures (MPa)	Temperature (°C)	Environment	H_2_S Content	Other Impurities	Test Period (h)	Corrosion Rate (mm/y)	Local Corrosion Rate (mm/y)	Corrosion Products	Reference
P110	10	80	water-saturated CO_2_ phase	50 ppmv		240	<0.2	0.52	FeCO_3,_ mackinawite, Cr(OH)_3_	[57]
3Cr							>0.4	0.84		
316L							0	0	FeCO_3_, mackinawite	
P110			CO_2_-saturated water phase				10.37	<6	FeCO_3_, mackinawite, pyrrhotie, Cr(OH)_3_	
3Cr							2.71	<2		
316L							<0.02	>0.024	FeCO_3_, Cr(OH)_3_, nickel sulfide, mackinawite	
X65	10	80	water-saturated CO_2_ phase	0		240	0.17	0.29	FeCO_3_	[58]
				50 ppmv			0.24	0.48	FeCO_3_, Fe_1−x_S, Fe_1+x_S,	
			CO_2_-saturated NaCl solution	0 ppmv			8.46	9.19	FeCO_3_	
				50 ppmv			15.48	2.45	FeCO_3_, FeS, Fe_1−x_S	
Q125	14.2	140	water-saturated CO_2_ phase	1.33 KPa		168	0.015		FeCO_3_, Fe_1−x_S, Cr(OH)_3_	[59]
				7.24 KPa			0.041			
			CO_2_-saturated formation water phase	1.33 KPa			0.081			
				7.24 KPa			0.051			
X65	8	50	water-saturated CO_2_ phase	0.08 bar	SO_2_ 0.08 bar, O_2_ 0.08 bar	1.5	>20		FeS, FeSO_4_∙xH_2_O, FeCO_3_	[60]
						72	2.57		FeS, FeSO_4_∙xH_2_O, FeCO_3_, FeOOH, S	

**Table 4:** Corrosion behavior of pipeline steel with various SO_2_ content in supercritical CO_2_.

Materials	Pressures (MPa)	Temperature (°C)	Environment	SO_2_ Content	Other Impurities	Test Period (h)	Corrosion Rate (mm/y)	Corrosion Products	Reference
X70	10	50	water-saturated CO_2_ phase	0 ppm		120	0.014	FeSO_3_, FeCO_3_,	[47]
				200 ppm			0.269		
				600 ppm			0.345		
				1000 ppm			0.423		
				1000 ppm	O_2_ 1000 ppm		0.842	FeSO_3_, FeSO_4_, FeCO_3_, FeOOH	
X70	10	40	water-saturated CO_2_ phase	500 ppmv		48	1.1	FeSO_3_∙2H_2_O, FeCO_3_, FeOOH	[51]
					O_2_ 200 ppmv		1.24	FeSO_3_∙2H_2_O, FeSO_3_∙3H_2_O, FeCO_3_, Fe_2_O_3_	
					O_2_ 1000 ppmv		0.6		
X70	10	50	water-saturated CO_2_ phase	0.2 mol%	O_2_ 1000 ppm	288	0.2	FeSO_4_∙7H_2_O,	[61]
				0.7 mol%			0.6–0.7	FeSO_4_∙4H_2_O, FeSO_3_∙3H_2_O	
				1.4 mol%			0.75–0.9	FeSO_4_∙4H_2_O, α-FeOOH	
				2.0 mol%			0.9	FeSO_4_∙4H_2_O	
X80	8	35	CO_2_-saturated water phase	0		48	1.67	FeCO_3_, Fe_2_O_3_	[63]
				5%			3.08	FeCO_3_, FeSO_3_∙xH_2_O, FeS	
X65	10	50	water-saturated CO_2_ phase	0		120	0.015	FeCO_3_,	[64]
				1000 ppmv			0.469	FeSO_3_ FeSO_3_∙xH_2_O, FeSO_4_∙4H_2_O, FeCO_3_, Fe_2_O_3_∙H_2_O	
X65	8	35	water-saturated CO_2_ phase	0		72	0.1	FeCO_3_	[65]
				50 ppm			0.37	FeCO_3_ FeSO_3_∙3H_2_O	
				100 ppm			0.72		
carbon steel	9.5	60	CO_2_ phase	0	H_2_O 1000 ppm	1512	0.00352	FeCO_3_, α-FeOOH, Fe_3_O_4_	[66]
				0.50%			0.3375	γ-FeOOH	
				1.50%			0.5–0.6		
				5%			1.396	FeCO_3_, FeSO_3_∙3H_2_O	

## Data Availability

No new data were created or analyzed in this study.

## References

[1] Ren S., Jiao X., Zheng D., Zhang Y., Xie H., Guo Z. (2024). Demand and Fluctuation Range of China’s Coal Production under the Dual Carbon Target. Energy Rep..

[2] Wen W., Su Y., Tang Y., Zhang X., Hu Y., Ben Y., Qu S. (2024). Evaluating Carbon Emissions Reduction Compliance Based on “dual Control” Policies of Energy Consumption and Carbon Emissions in China. J. Environ. Manag..

[3] Zhu H., Cao S., Su Z., Zhuang Y. (2024). China’s Future Energy Vision: Multi-Scenario Simulation Based on Energy Consumption Structure under Dual Carbon Targets. Energy.

[4] Zhang W., Chen X., Tian J. (2024). The Evolutionary Analysis of Investment in CCS-EOR under Dual Carbon Target—From the Perspective of Multi-Agent Involvement. Int. J. Greenh. Gas Control.

[5] Dou L., Sun L., Lyu W., Wang M., Gao F., Gao M., Jiang H. (2023). Trend of Global Carbon Dioxide Capture, Utilization and Storage Industry and Challenges and Countermeasures in China. Pet. Explor. Dev..

[6] Wang W., Guang Y., Liu W., Shen K., Huffman M., Wang Q. (2023). Experimental Investigation of Stress Corrosion on Supercritical CO_2_ Transportation Pipelines against Leakage for CCUS Applications. Energy Rep..

[7] Global Status of CCS 2024. https://www.globalccsinstitute.com/resources/global-status-report/.

[8] Kim T.W., Yoon H.C., Lee J.Y. (2024). Review on Carbon Capture and Storage (CCS) from Source to Sink; Part 1: Essential Aspects for CO_2_ Pipeline Transportation. Int. J. Greenh. Gas Control.

[9] Peletiri S.P., Rahmanian N., Mujtaba I.M. (2018). CO_2_ Pipeline Design: A Review. Energies.

[10] Miao L., Feng L., Ma Y. (2025). Comprehensive Evaluation of CCUS Technology: A Case Study of China’s First Million-Tonne CCUS-EOR Project. Environ. Impact Assess. Rev..

[11] Yang L., Rui W., Zhao Q.M., Zhou Y.L., Xin F., Xue Z.J. (2024). CO_2_-Enhanced Oil Recovery with CO_2_ Utilization and Storage: Progress and Practical Applications in China. Unconv. Resour..

[12] Sarrade S., Feron D., Rouillard F., Perrin S., Robin R., Ruiz J.-C., Turc H.-A. (2017). Overview on Corrosion in Supercritical Fluids. J. Supercrit. Fluids.

[13] Bacca K.R.G., Lopes N.F., Batista G.d.S., dos Santos C.A., Costa E.M.d. (2022). Electrochemical, Mechanical, and Tribological Properties of Corrosion Product Scales Formed on X65 Steel under CO_2_ Supercritical Pressure Environments. Surf. Coat. Technol..

[14] Eldevik F., Graver B., Torbergsen L.E., Saugerud O.T. (2009). Development of a Guideline for Safe, Reliable and Cost Efficient Transmission of CO_2_ in Pipelines. Energy Procedia.

[15] Wang Z.M., Song G.L., Zhang J. (2019). Corrosion Control in CO_2_ Enhanced Oil Recovery From a Perspective of Multiphase Fluids. Front. Mater..

[16] Luo X., Wang M., Oko E., Okezue C. (2014). Simulation-Based Techno-Economic Evaluation for Optimal Design of CO_2_ Transport Pipeline Network. Appl. Energy.

[17] El-Kady A.H., Amin M.T., Khan F., El-Halwagi M.M. (2024). Analysis of CO_2_ Pipeline Regulations from a Safety Perspective for Offshore Carbon Capture, Utilization, and Storage (CCUS). J. Clean. Prod..

[18] Nath F., Mahmood M.N., Yousuf N. (2024). Recent Advances in CCUS: A Critical Review on Technologies, Regulatory Aspects and Economics. Geoenergy Sci. Eng..

[19] Li C., Xiang Y., Song C., Ji Z. (2019). Assessing the Corrosion Product Scale Formation Characteristics of X80 Steel in Supercritical CO_2_-H_2_O Binary Systems with Flue Gas and NaCl Impurities Relevant to CCUS Technology. J. Supercrit. Fluids.

[20] Li Q.K., Kutana A., Penev E.S., Yakobson B.I. (2022). Iron Corrosion in the “Inert” Supercritical CO_2_, Ab Initio Dynamics Insights: How Impurities Matter. Matter.

[21] Hua Y., Barker R., Charpentier T., Ward M., Neville A. (2015). Relating Iron Carbonate Morphology to Corrosion Characteristics for Water-Saturated Supercritical CO_2_ Systems. J. Supercrit. Fluids.

[22] Zhang G.A., Zeng Y., Guo X.P., Jiang F., Shi D.Y., Chen Z.Y. (2012). Electrochemical Corrosion Behavior of Carbon Steel under Dynamic High Pressure H_2_S/CO_2_ Environment. Corros. Sci..

[23] Xiang Y., Xu M., Choi Y.S. (2017). State-of-the-Art Overview of Pipeline Steel Corrosion in Impure Dense CO_2_ for CCS Transportation: Mechanisms and Models. Corros. Eng. Sci. Technol..

[24] (2005). Standard Recommended Practice: Preparation, Installation, Analysis, and Interpretation of Corrosion Coupons in Oilfield Operations.

[25] Xiang Y., Song C., Li C., Yao E., Yan W. (2020). Characterization of 13Cr Steel Corrosion in Simulated EOR-CCUS Environment with Flue Gas Impurities. Process Saf. Environ. Prot..

[26] Ma G. (2024). Corrosion Behavior of N80 Steel in Underground Supercritical CO_2_ Environments. Chem. Tech. Fuels Oils.

[27] Simonsen K.R., Hansen D.S., Pedersen S. (2024). Challenges in CO_2_ Transportation: Trends and Perspectives. Renew. Sustain. Energy Rev..

[28] Slavchov R.I., Iqbal M.H., Faraji S., Madden D., Sonke J., Clarke S.M. (2024). Corrosion Maps: Stability and Composition Diagrams for Corrosion Problems in CO_2_ Transport. Corros. Sci..

[29] Li Y., Wang W., Chen Z.F., Li Y.X. (2023). Integrity Assessment of Supercritical CO_2_ Transport Pipelines. Geoenergy Sci. Eng..

[30] Fu L., Ren Z., Si W., Ma Q., Huang W., Liao K., Huang Z., Wang Y., Li J., Xu P. (2022). Research Progress on CO_2_ Capture and Utilization Technology. J. CO2 Util..

[31] Jayakumar A., Gomez A., Mahinpey N. (2016). Post-Combustion CO_2_ Capture Using Solid K_2_CO_3_: Discovering the Carbonation Reaction Mechanism. Appl. Energy.

[32] Li K., Zeng Y. (2023). Advancing the Mechanistic Understanding of Corrosion in Supercritical CO_2_ with H_2_O and O_2_ Impurities. Corros. Sci..

[33] Yan T., Xu L.-C., Zeng Z.-X., Pan W.-G. (2024). Mechanism and Anti-Corrosion Measures of Carbon Dioxide Corrosion in CCUS: A Review. iScience.

[34] Cui G., Yang Z., Liu J., Li Z. (2019). A Comprehensive Review of Metal Corrosion in a Supercritical CO_2_ Environment. Int. J. Greenh. Gas Control.

[35] Jiang X., Qu D., Song X., Liu X., Zhang Y. (2019). Critical Water Content for Corrosion of X65 Mild Steel in Gaseous, Liquid and Supercritical CO_2_ Stream. Int. J. Greenh. Gas Control.

[36] Hua Y., Barker R., Neville A. (2015). Comparison of Corrosion Behaviour for X-65 Carbon Steel in Supercritical CO_2_-Saturated Water and Water-Saturated/Unsaturated Supercritical CO_2_. J. Supercrit. Fluids.

[37] Liu G.Y., Fan X.X., Wang C.L., Zhao X.F., Meng L., Hu Q.H., Li Y.X. (2025). Study on the pitting corrosion behavior of X65 steel in supercritical and dense-phase CO_2_ based on in-situ electrochemical noise measurement. Process Saf. Environ. Prot..

[38] Xiang Y., Wang Z., Yang X., Li Z., Ni W. (2012). The Upper Limit of Moisture Content for Supercritical CO_2_ Pipeline Transport. J. Supercrit. Fluids.

[39] Liu J., Yao D., Chen K., Wang C., Sun C., Pan H., Meng F., Chen B., Wang L. (2023). Effect of H_2_O Content on the Corrosion Behavior of X52 Steel in Supercritical CO_2_ Streams Containing O_2_, H_2_S, SO_2_ and NO_2_ Impurities. Energies.

[40] Sun C., Sun J., Liu S., Wang Y. (2018). Effect of Water Content on the Corrosion Behavior of X65 Pipeline Steel in Supercritical CO_2_-H_2_O-O_2_-H_2_S-SO_2_ Environment as Relevant to CCS Application. Corros. Sci..

[41] Xu M., Li W., Zhou Y., Yang X., Wang Z., Li Z. (2016). Effect of Pressure on Corrosion Behavior of X60, X65, X70, and X80 Carbon Steels in Water-Unsaturated Supercritical CO_2_ Environments. Int. J. Greenh. Gas Control.

[42] Hua Y., Barker R., Neville A. (2015). The Effect of O_2_ Content on the Corrosion Behaviour of X65 and 5Cr in Water-Containing Supercritical CO_2_ Environments. Appl. Surf. Sci..

[43] Xu M., Zhang Q., Wang Z., Liu J., Li Z. (2016). Effect of High-Concentration O_2_ on Corrosion Behavior of X70 Steel in Water-Containing Supercritical CO_2_ with SO_2_. Corrosion.

[44] Barker R., Hua Y., Neville A. (2017). Internal Corrosion of Carbon Steel Pipelines for Dense-Phase CO_2_ Transport in Carbon Capture and Storage (CCS)—A Review. Int. Mater. Rev..

[45] (2023). Standard Recommended Practice: Preparation, Installation, Analysis, and Interpretation of Corrosion Coupons in Hydrocarbon Operations.

[46] He L., Zhang Q., Chen W., Wang Y., Wang M., Huang Y., Xu Y. (2024). Unraveling Short-Term O_2_ Contamination on under Deposit Corrosion of X65 Pipeline Steel in CO_2_ Saturated Solution. Corros. Sci..

[47] Sun J., Sun C., Wang Y. (2018). Effects of O_2_ and SO_2_ on Water Chemistry Characteristics and Corrosion Behavior of X70 Pipeline Steel in Supercritical CO_2_ Transport System. Ind. Eng. Chem. Res..

[48] Basilico E., Marcelin S., Mingant R., Kittel J., Fregonese M., Barker R., Owen J., Neville A., Ropital F. (2022). Effect of O_2_ Contamination on Carbon Steel Pseudo-Passive Scales in CO_2_ Aqueous Solutions. Corros. Sci..

[49] Li Y.Y., Jiang Z.N., Zhang Q.H., Lei Y., Wang X., Zhang G.A. (2022). Unveiling the Influential Mechanism of O_2_ on the Corrosion of N80 Carbon Steel under Dynamic Supercritical CO_2_ Conditions. Corros. Sci..

[50] Tang Y., Guo X.P., Zhang G.A. (2017). Corrosion Behaviour of X65 Carbon Steel in Supercritical-CO_2_ Containing H_2_O and O_2_ in Carbon Capture and Storage (CCS) Technology. Corros. Sci..

[51] Wang W., Shen K., Tang S., Shen R., Parker T., Wang Q. (2019). Synergistic Effect of O_2_ and SO_2_ Gas Impurities on X70 Steel Corrosion in Water-Saturated Supercritical CO_2_. Process Saf. Environ. Prot..

[52] Visser E., Hendriks C., Barrio M., Mølnvik M.J., Koeijer G., Liljemark S., Le Gallo Y. (2008). Dynamis CO_2_ Quality Recommendations. Int. J. Greenh. Gas Control.

[53] Li K., Zeng Y., Luo J.L. (2021). Influence of H_2_S on the General Corrosion and Sulfide Stress Cracking of Pipelines Steels for Supercritical CO_2_ Transportation. Corros. Sci..

[54] Sun C., Ding T., Sun J., Lin X., Zhao W., Chen H. (2023). Insights into the Effect of H_2_S on the Corrosion Behavior of N80 Steel in Supercritical CO_2_ Environment. J. Mater. Res. Technol..

[55] Sun C., Sun J., Luo J.L. (2020). Unlocking the Impurity-Induced Pipeline Corrosion Based on Phase Behavior of Impure CO_2_ Streams. Corros. Sci..

[56] Jou F., Carroll J., Mather A., Otto F. (1993). Solubility of Mixtures of Hydrogen-Sulfide and Carbon-Dioxide in Aqueous N-Methyldiethanolamine Solutions. J. Chem. Eng. Data.

[57] Wei L., Pang X., Gao K. (2016). Corrosion of Low Alloy Steel and Stainless Steel in Supercritical CO_2_/H_2_O/H_2_S Systems. Corros. Sci..

[58] Wei L., Pang X., Gao K. (2016). Effect of Small Amount of H_2_S on the Corrosion Behavior of Carbon Steel in the Dynamic Supercritical CO_2_ Environments. Corros. Sci..

[59] Wang Y., Wang B., He S., Zhang L., Xing X., Li H., Lu M. (2022). Unraveling the Effect of H_2_S on the Corrosion Behavior of High Strength Sulfur-Resistant Steel in CO_2_/H_2_S/Cl^−^ Environments at Ultra High Temperature and High Pressure. J. Nat. Gas Sci. Eng..

[60] Sun C., Liu J., Sun J., Lin X., Wang Y. (2021). Probing the Initial Corrosion Behavior of X65 Steel in CCUS-EOR Environments with Impure Supercritical CO_2_ Fluids. Corros. Sci..

[61] Xiang Y., Wang Z., Xu C., Zhou C., Li Z., Ni W. (2011). Impact of SO_2_ Concentration on the Corrosion Rate of X70 Steel and Iron in Water-Saturated Supercritical CO_2_ Mixed with SO_2_. J. Supercrit. Fluids.

[62] Hua Y., Barker R., Neville A. (2015). The Influence of SO_2_ on the Tolerable Water Content to Avoid Pipeline Corrosion during the Transportation of Supercritical CO_2_. Int. J. Greenh. Gas Control.

[63] Li C., Xiang Y., Wang R.T., Yuan J., Xu Y.H., Li W.G., Zheng Z.G. (2023). Exploring the inffuence of ffue gas impurities on the electrochemical corrosion mechanism of X80 steel in a supercritical CO_2_-saturated aqueous environment. Corros. Sci..

[64] Sun C., Sun J., Wang Y., Lin X., Li X., Cheng X., Liu H. (2016). Synergistic Effect of O_2_, H_2_S and SO_2_ Impurities on the Corrosion Behavior of X65 Steel in Water-Saturated Supercritical CO_2_ System. Corros. Sci..

[65] Jiang X., Song X.L., Yu C., Xie H., Qu D.R., Hua J. (2025). Investigation of SO_2_-induced corrosion of X65 steel in gaseous, liquid, and supercritical CO_2_ environments through experimental and thermodynamic approaches. J. Supercrit. Fluids.

[66] Mahlobo M.G.R., Premlall K., Olubambi P.A. (2022). Effect of Exposure Time with SO_2_ as an Impurity on the Corrosion Behaviour of Pipeline Steel in CCS Transportation. Corros. Eng. Sci. Technol..

[67] Morland B., Truls N., Tjelta M., Svenningsen G. (2019). Effect of SO_2_, O_2_, NO_2_, and H_2_O Concentrations on Chemical Reactions and Corrosion of Carbon Steel in Dense Phase CO_2_. Corrosion.

[68] Dugstad A., Halseid M., Morland B. (2013). Effect of SO_2_ and NO_2_ on Corrosion and Solid Formation in Dense Phase CO_2_ Pipelines. Energy Procedia.

[69] Sun C., Wang Y., Sun J., Lin X., Li X., Liu H., Cheng X. (2016). Effect of Impurity on the Corrosion Behavior of X65 Steel in Water-Saturated Supercritical CO_2_ System. J. Supercrit. Fluids.

[70] Sun C., Yan X., Sun J., Pang J., Zhao W., Lin X. (2022). Unraveling the Effect of O_2_, NO_2_ and SO_2_ Impurities on the Stress Corrosion Behavior of X65 Steel in Water-Saturated Supercritical CO_2_ Streams. Corros. Sci..

[71] Kairy S.K., Zhou S., Turnbull A., Hinds G. (2023). Corrosion of Pipeline Steel in Dense Phase CO_2_ Containing Impurities: A Critical Review of Test Methodologies. Corros. Sci..

[72] Honarvar Nazari M., Allahkaram S.R., Kermani M.B. (2010). The Effects of Temperature and pH on the Characteristics of Corrosion Product in CO_2_ Corrosion of Grade X70 Steel. Mater. Des..

[73] Sui P., Sun J., Hua Y., Liu H., Zhou M., Zhang Y., Liu J., Wang Y. (2018). Effect of Temperature and Pressure on Corrosion Behavior of X65 Carbon Steel in Water-Saturated CO_2_ Transport Environments Mixed with H_2_S. Int. J. Greenh. Gas Control.

[74] Hua Y., Barker R., Neville A. (2014). Effect of Temperature on the Critical Water Content for General and Localised Corrosion of X65 Carbon Steel in the Transport of Supercritical CO_2_. Int. J. Greenh. Gas Control.

[75] Wang H., Zhang L., Gan M., Su X., Wang Y., Xue Q., Mei K., Fu X. (2024). Temperature and Reaction Time’s Effects on N80 Steel Corrosion Behavior in Supercritical CO_2_ and Formation Water Environments. Appl. Sci..

[76] Azevedo Rodrigues T.R.S., Marcolino J.B., Moraes M.K., Lopes N.F., Costa E.M. (2024). Influence of CO_2_ Subcritical and Supercritical Pressures on the Protective Properties of Corrosion Product Scales Formed on X65 Steel. J. Supercrit. Fluids.

[77] Li W., Pots B.F.M., Brown B., Kee K.E., Nesic S. (2016). A Direct Measurement of Wall Shear Stress in Multiphase Flow—Is It an Important Parameter in CO_2_ Corrosion of Carbon Steel Pipelines?. Corros. Sci..

[78] Wei L., Pang X., Gao K. (2018). Effect of Flow Rate on Localized Corrosion of X70 Steel in Supercritical CO_2_ Environments. Corros. Sci..

[79] Zeng L., Lv T., Chen H., Ma T., Fang Z., Shi J. (2023). Flow Accelerated Corrosion of X65 Steel Gradual Contraction Pipe in High CO_2_ Partial Pressure Environments. Arab. J. Chem..

[80] Li Y., Liu D., Zhu G., Zhang G. (2020). Effects of temperature and flow rate on the corrosion behavior of N80 carbon steel in supercritical CO_2_ environment (China). Surf. Technol..

[81] Wang Y., Wang B., Xing X., He S., Zhang L., Lu M. (2022). Effects of Flow Velocity on the Corrosion Behaviour of Super 13Cr Stainless Steel in Ultra-HTHP CO_2_-H_2_S Coexistence Environment. Corros. Sci..

[82] Wang X., Yang P., Li R., Tong G., Chen J., Wang Y. (2024). Investigation of Supercritical CO_2_ Corrosion Behavior of X80 Carbon Steel in Pipelines: An in Situ Experimental and DFT Study. J. Supercrit. Fluids.

[83] Hua Y., Jonnalagadda R., Zhang L., Neville A., Barker R. (2017). Assessment of General and Localized Corrosion Behavior of X65 and 13Cr Steels in Water-Saturated Supercritical CO_2_ Environments with SO_2_/O_2_. Int. J. Greenh. Gas Control.

[84] Wang L.W., Liu Z.Y., Cui Z.Y., Du C.W., Wang X.H., Li X.G. (2014). In Situ Corrosion Characterization of Simulated Weld Heat Affected Zone on API X80 Pipeline Steel. Corros. Sci..

[85] Sharma S.K., Maheshwari S. (2017). A Review on Welding of High Strength Oil and Gas Pipeline Steels. J. Nat. Gas Sci. Eng..

[86] Zhang S., Yang F., Jia H., Chen C., Feng Q., Dai L., Zhang Z. (2024). Study on the Effect of Microstructure on Toughness Dispersion of X70 Steel Girth Weld. Eng. Fail. Anal..

[87] Li J., Wang D., Xie F. (2022). Failure Analysis of CO_2_ Corrosion of Natural Gas Pipeline under Flowing Conditions. Eng. Fail. Anal..

[88] Ding H.X., Xiang Y., Lu W.P., Yan K., Ren J.R., Yan W., Yao E.D., Zhao X.H. (2024). Selective adsorption and corrosion mechanism of SO_2_ and its hydrates on X65 welded joints steel in CO_2_-saturated aqueous solution. Corros. Sci..

[89] Bai F., Ding H., Tong L., Pan L. (2020). Microstructure and Properties of the Interlayer Heat-Affected Zone in X80 Pipeline Girth Welds. Prog. Nat. Sci. Mater. Int..

[90] Lu Y., Xu L. (2020). Early Corrosion Stage of Welded Carbon Steel Joints in CO_2_-Saturated Oilfield Water. Mater. Test..

[91] Yan Y., Deng H., Xiao W., Ou T., Cao X. (2020). Corrosion Behaviour of X80 Pipeline Steel Welded Joint in H_2_O-Saturated Supercritical-CO_2_ Environment. Int. J. Electrochem. Sci..

[92] Yao B., Gao Q., Zhang Z., Yan Y., Deng H., Lan W. (2022). Effect of Microstructure of Welded Joint of X80 pipeline Steel on Its Corrosion Behaviors in Supercritical CO_2_ (China). Corros. Prot..

